# Cascade Genetic Testing for Hereditary Cancer Predisposition: Characterization of Patients in a Catchment Area of Southern Italy

**DOI:** 10.3390/genes16070795

**Published:** 2025-06-30

**Authors:** Anna Bilotta, Elisa Lo Feudo, Valentina Rocca, Emma Colao, Francesca Dinatolo, Serena Marianna Lavano, Paola Malatesta, Lucia D’Antona, Rosario Amato, Francesco Trapasso, Nicola Perrotti, Giuseppe Viglietto, Francesco Baudi, Rodolfo Iuliano

**Affiliations:** 1 Medical Genetics Unit, Renato Dulbecco University Hospital, 88100 Catanzaro, Italy; annabilotta@unicz.it (A.B.); elisa.lofeudo@studenti.unicz.it (E.L.F.); valentina.rocca@unicz.it (V.R.); colaoemma@unicz.it (E.C.); francesca.dinatolo@studenti.unicz.it (F.D.); serenamarianna.lavano@studenti.unicz.it (S.M.L.); p.malatesta@unicz.it (P.M.); dantona@unicz.it (L.D.); rosario.amato@unicz.it (R.A.); trapasso@unicz.it (F.T.); perrotti@unicz.it (N.P.); viglietto@unicz.it (G.V.); baudi@unicz.it (F.B.); 2 Department of Clinical and Experimental Medicine, Campus S. Venuta, University Magna Graecia of Catanzaro, 88100 Catanzaro, Italy; 3 Department of Health Sciences, Campus S. Venuta, University Magna Graecia of Catanzaro, 88100 Catanzaro, Italy

**Keywords:** pathogenic variants, hereditary cancer predisposition syndrome, cascade genetic testing, genetic testing uptake

## Abstract

**Background**: The national guidelines, informed by evidence from the National Institutes of Health (NIH), define the criteria for genetic testing of BRCA1/2 and other genes associated with Hereditary Breast and Ovarian Cancer (HBOC) and Lynch Syndrome (LS). When a germline pathogenic variant (PV) is identified in an index case, clinical recommendations advise informing at-risk relatives about the availability of predictive genetic testing, as early identification of carriers allows for timely implementation of preventive measures. **Methods**: This retrospective observational study examined data collected between 2017 and 2024 at the Medical Genetics Unit of the “Renato Dulbecco” University Hospital in Catanzaro, Italy. The analysis focused on trends in the identification of individuals carrying PVs in cancer predisposition genes (CPGs) and the subsequent uptake of cascade genetic testing (CGT) among their family members. **Results**: Over the study period, from 116 probands were performed 257 CGTs on 251 relatives. A notable reduction of approximately ten years in median age was observed, 39% were found to carry familial mutation and were referred to personalized cancer prevention programs. Among these, 62% accessed Oncological Genetic Counselling (CGO) within one year of the proband’s diagnosis, suggesting effective communication and outreach. **Conclusions**: The findings highlight the critical role of effective CGO and intrafamilial communication in hereditary cancer prevention. The identification of PVs, followed by timely CGTs and implementation of preventive strategies, significantly contributes to early cancer risk management. Periodic monitoring of CGT uptake and outcome trends, as demonstrated in this study, is essential to refine and optimize genetic services and public health strategies.

## 1. Introduction

HCPS (Hereditary Cancer Predisposition Syndrome) was responsible for only 5–10% of cancer cases. More recent studies, however, have shown that this percentage can reach 17.5% [1]. HCPS is due to the presence of pathogenic and probably pathogenic germline variants (PVs) in tumor suppressors and oncogenes [2]. Most cases of HCPS are characterized by autosomal dominant inheritance and include *Lynch* syndrome, Li-Fraumeni syndrome, HBOC, and other cancers [3,4].

Cancer-predisposing genes are characterized by different tumor-specific penetrance (high, moderate, or low) and are therefore classified into distinct groups based on their association with the risk of developing specific cancers. Penetrance was defined as low, moderate, or high based on a 0% to 20%, 20% to 50%, or 50% to 100% probability of developing a particular type of tumor, respectively [5]. For some genes, there is insufficient evidence regarding their association with the risk of developing cancer. Furthermore, this classification is continuously updated based on clinical and experimental evidence [6]. A gene with high penetrance has a high probability of being expressed phenotypically in a carrier of a PV. These genes are at high risk because, if mutated, they significantly increase the risk of developing cancer compared to the general population [6]. Some of the high-risk genes include *BRCA1, BRCA2, TP53,* and *PTEN* [7]. *PALB2* is considered a high-to-moderate risk gene and is particularly elevated in breast cancer [8]. Genes with moderate penetrance, on the other hand, are associated with a moderate risk of two to four times compared to the general population [6]. Moderate-risk genes include *CHEK2, ATM, RAD51C, RAD51D*, and *BARD1* [7,8]. Low penetrance indicates a low probability of developing a tumor; therefore, genes with low penetrance are associated with a low risk, although some pathogenic variants may confer a higher risk for a certain type of cancer [5]. However, the probability of developing cancer varies within each individual carrier (even within the same family), which is likely attributable to other yet to be identified factors, including epigenetic modification or environmental factors that are influencing cancer penetrance. Breast cancer risk has also been discovered to be influenced by polygenic risk scores (PRS), which are a collection of single-nucleotide polymorphisms (relatively common genetic variants) that together serve to either increase or decrease risk. Individually, these genetic variants have little impact [8]. Other factors can influence cancer development. Microbial roles in cancer formation, diagnosis, prognosis, and treatment have been disputed for centuries. Recent studies have provocatively claimed that bacteria, viruses, and/or fungi are pervasive among cancers, key actors in cancer immunotherapy, and engineerable to treat metastases [9]. For example, the gastrointestinal microbiota plays a significant role in colorectal carcinogenesis [10].

Nowadays, the recognition of individuals carrying germline variants predisposing them to cancer has been facilitated by analysis using Next Generation Sequencing (*NGS*), which utilizes multigene panels. The latter includes genes with high and moderate risk, allowing for a more detailed analysis [2]. In fact, in the past, genetic tests for HBOC were mostly based on the analysis of the highly penetrant genes *BRCA1/2*. In recent years, a significant association has been observed between breast, ovarian, endometrial, stomach, and colon cancers and genes such as *PALB2, ATM, MSH2, MLH1, PMS2, TP53, CDH1*, and *STK11* [11]. The NCCN evidence-based guidelines, professional practice guidelines, published scientific literature, and test registries periodically update the cancer panels with their relative included genes. Such panels are usually designed as pan-cancer panels and contain many genes that must be thoroughly considered by laboratories during test development. The scientific evidence for the inclusion of specific genes in a panel construction by laboratories needs to be documented in the validation protocol [12].

The identification of a germline pathogenic or probably pathogenic variant in the index case, through *NGS* sequencing, facilitates the initiation of the Cascade Genetic Testing (CGT). It consists of extending genetic testing to the at-risk relatives of germline PV carriers, by *Sanger* sequencing, to adopt a management plan aimed at early diagnosis and risk reduction in positive cases (predictive test) [13,14,15]. The choice of strategy to undertake must be discussed with at-risk family relatives during post-test counseling after assessing the risks and benefits [15]. Therefore, the CGT could play an important role as a means of cancer prevention for individuals with hereditary risk [16]. This represents a benefit for carriers, but also for the health system, as it offers the opportunity to reduce cancer incidence, morbidity, and mortality in a cost-effective manner [17].

In Italy, subjects at high risk of hereditary cancer predisposition syndrome undergo oncology genetic counseling regulated according to the international guidelines available worldwide [7,14,15,18,19,20]. However, these guidelines lack more detailed recommendations regarding standardized procedures to increase disclosure in at-risk families; nonetheless, it is often challenging not only to construct the pedigree but also to transmit information to the family members mediated by the proband [21]. Specifically, there are no specific guidelines, but in general clinical practice, it is expected that geneticists exhaustively explain the meaning associated with the variant found to the proband who tested positive, encouraging them to transmit the information received as quickly as possible to the closest family members to induce them to request genetic counseling themselves [22,23].

Within the Italian National Health Service (called *Servizio Sanitario Nazionale*, SSN), individuals belonging to the high-risk category for hereditary cancer syndrome, as well as their family members, receive Oncological Genetic Counselling (CGO) according to the guidelines set by the Italian Association of Medical Oncology (AIOM) and by the Italian Society of Human Genetics (SIGU), which are similar to the NCCN guidelines established at an international level within the global national oncology network. According to these, when a pathogenic or probably pathogenic variant is identified in the index case, targeted genetic testing (i.e., searching for the familial variant) can be extended to other family members who wish to undergo it, starting from the age of 18 [10,11,14]. The Medical Genetics Unit of the “Renato Dulbecco” University Hospital receives and adopts the AIOM/SIGU guidelines and provides the CGO to adults. Only in sporadic cases did the CGO refer to minors, which is not included in this retrospective analysis. Particularly, when PVs in the APC gene or *TP53* gene are present, in the case of Li-Fraumeni syndrome, for a high risk of pediatric acute lymphoblastic leukemia. The selection criteria for the analysis of the APC gene, for example, include healthy relatives at risk from the age of 10–12 years, when there is a family history of classical form, as the development of polyps can be very early and require timely execution of preventive surgery; and in late adolescence in the presence of a family history of the attenuated form (AAAP) [18].

Despite its positive impact on public health, the uptake of CGT is approximately 30% [17,24,25,26,27,28]. The understanding and transmission of information learned during consultations are conditioned by many factors such as age, sex, ethnicity, religion, and intra-family relationships. Several studies have highlighted the tendency of probands to inform their children and parents more than distant relatives. The family environment certainly affects the proband’s choice to share the test results or not, just as it influences the family member’s decision to participate in counseling and undergo the test [22,29,30]. However, the disclosure of genetic information to family members is closely connected to the genetic counseling process, as it can be facilitated by providing consultants with easily understandable and accessible information. During the post-test counseling, it is important to evaluate different cases individually, giving personalized advice on the most appropriate way to transmit information. In this way, it is easier for family members to receive correct information, making them more informed when deciding. Conversely, inadequate CGO could lead to misunderstandings, worries, and negative impacts on the patient’s emotions by causing incorrect transmission of information [23,29,30].

The objective of this retrospective observational study is to verify the validity of the cascade testing process in a catchment area of patients from Southern Italy through the analysis of data collected between 2017 and 2024 in the Medical Genetics Unit of the “Renato Dulbecco” University Hospital in Catanzaro (Italy). This will allow for improved patient-geneticist communication and the adoption of new strategies that are useful for service provision.

## 2. Materials and Methods

### 2.1. Study Design and Setting

This is an observational, retrospective study that took place at the Medical Genetics Unit of the “Renato Dulbecco” University Hospital in Catanzaro, Italy.

The process of genetic counseling included in-person pre-test and post-test counseling, according to standard procedures and guidelines of AIOM-SIGU-NCCN (Associazione Italiana di Oncologia Medica-Società Italiana di Genetica Umana-National Comprehensive Cancer Network) [7,14,15,18,19,20]. A section of the genetic test disclosure session was dedicated to discussing the importance of the genetic test results for relatives and identifying at-risk family members eligible for the step of cascade testing.

### 2.2. Probands and Relatives

Between January 2017 and June 2024, 251 patients aged 18 years or older received CGO at the Medical Genetics Unit of the “Renato Dulbecco” University Hospital and were candidates for the cascade genetic tests (CGTs) based on the presence of a biological family member within the second-degree (if the closest relative(s) was/were deceased) of kinship diagnosed with a hereditary predisposition to the development of tumors. The characteristics and relationships of CGT patients and index cases are elucidated in Table S1. Individuals from both maternal and paternal branches of the family were included if no indication of PV segregation was available.

After the analysis by *Sanger* sequencing in the region of the gene containing the “known variant” of the index case, the presence and characterization of the identified germline genetic variants were evaluated. Patients were informed of the outcome of the examination: if negative, that they had a risk of developing tumors equal to that of the general population; if positive, for the identification of the familial pathogenic variant (PV), that there was a need to activate close surveillance, primary and secondary prevention, and risk reduction programs (where possible). Moreover, there was a need to extend the search for the same PV in close relatives for the identification of carriers, who are also at higher risk of developing cancer than the general population and therefore candidates for prevention and risk reduction programs. The flowchart for identifying patients affected by hereditary cancer predisposition syndrome (HCPS) is presented Figure S1. Some of the individuals that underwent CGT originated from the genealogic tree of the index cases identified as PV carriers by *NGS* analysis at the Medical Genetics Unit of Catanzaro between January 2017 and June 2023 [31,32]. The inclusion period for *NGS*-analyzed cancer patients ended in June 2023 to allow a minimum period of one year for cascade test requests by the family members enrolled in the study.

From the clinical records of index cases, we retrieved the following information: gender, date of birth, age at PV identification, type of cancer(s), genetic test result, and date of genetic test result disclosure. For probands who performed *NGS* in the Medical Genetics Unit of Catanzaro, we also retrieved cancer family history, age at cancer diagnosis, and branch of the family. For each relative, the following information was collected: gender, date of birth, date of genetic counseling, age at genetic test, degree of relationship with the proband, and genetic test result.

All subjects included in this study signed a consent form. This study was approved by the “Comitato Etico Territoriale Regione Calabria” (Protocol n. 5 of 11 January 2024).

### 2.3. Data Analysis

Descriptive statistics included frequencies and percentages for categorical variables and means. The Chi-square test was used to compare differences among categorical variables, while the Student’s *t*-test was employed to compare quantitative data. Significance was defined as *p* < 0.05.

## 3. Results

### 3.1. Characterization of Patients

The analysis of the medical records of patients undergoing genetic testing showed that a total of 116 probands possessed familial pathogenic variants (PVs). Of these, 79 had undergone next-generation sequencing (*NGS*) analysis to determine the hereditary nature of the tumor, with 25 at the Medical Genetics Unit of Catanzaro (NGS p/o “*R. Dulbecco*”) and 54 in other healthcare facilities (NGS p/o other facilities). The remaining 37 probands were healthy carriers who had undergone *Sanger* sequencing of the familial pathogenic variant (*Sanger* report).

The 251 patients who performed the cascade test (CGT patients) included 72 family members of probands identified by *NGS* at the *“Renato Dulbecco” University Hospital*, 106 relatives of probands identified by *NGS* in other facilities, and 73 kin of probands with a *Sanger* report. Genetic tests facilitated the identification of 98 carriers of the familial PV (HCPS patients), which accounted for 39% of the total, with 25% derived from index cases identified by *NGS* at the Medical Genetics Unit of Catanzaro (Table 1).

(Index cases) Probands; (CGT patients) cascade Genetic Test patients; (HCPS patients) Hereditary Cancer Predisposition Syndrome patients; (NGS p/o “R. Dulbecco”) patients that performed NGS analysis at the Medical Genetics Unit of Catanzaro; (NGS p/o other facilities) patients that performed NGS analysis in other facilities; (Sanger report) patients that performed traditional sequencing for the presence of the familial pathogenetic variant.

### 3.2. Evaluation of the Perception of Cancer Risk and the Gender of Patients

**Figure 1:**
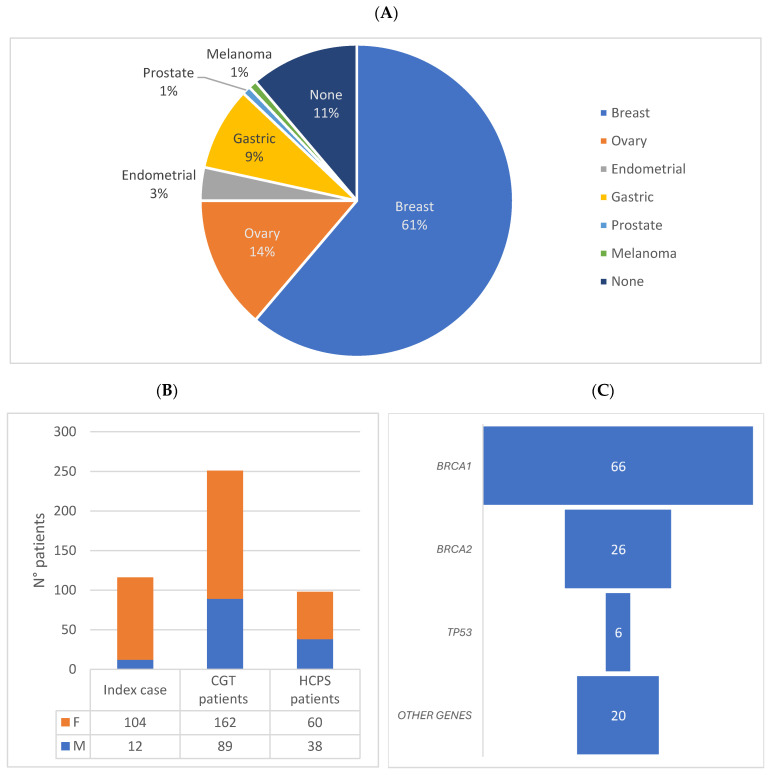
Characterization of the index cases, the CGT patients, and the HCPS patients. (**A**) Tumor type of the index cases. (**B**) Gender evaluation. (**C**) Gene localization of familial PVs.

To assess the perception of the risk of developing tumors among patients who performed the cascade test, the study examined the characteristics of the index cases, the CGT patients, and the familial PVs. The subdivision of the index cases according to tumors showed that 61% of the 116 probands had “breast” cancer (n = 70 women and n = 1 man), while 39% had different cancers (“ovary” n = 16; “gastric” n = 10; “endometrial” n = 4; “ prostate “ and “melanoma” n = 1; and “other cancers” n = 13) (Figure 1A). According to the primary proband tumors, the analysis based on the gender of the 116 probands reveals that there were 104 women and 12 men. The 251 family members who had undergone the genetic test comprised 162 females and 89 males. A greater number of female patients (162/251) requested the cascade test compared to male patients (89/251) (*p* < 0.00001, chi-square test). The HCPS patients included 60 women and 38 men (Figure 1B). Regarding the gene in which the familial genetic variants were located, the major perception of the risk of developing a tumor by the family members, and the effective information transfer by the index case, 66 and 26 familial pathogenic variants were located in the *BRCA1* and *BRCA2* genes (corresponding to 56% and 22% of the total), respectively, 6 PVs were in the *TP53* gene (5%), and 20 were in other genes (5 in the *MSH6* gene, 4 in the *ATM* gene, 2 in the *CHEK2*, *MLH1*, and *MSH2* genes, and finally 1 in the *PALB2, APC, RAD51C, BRIP1*, and *MITF* genes) (Figure 1C).

### 3.3. Evaluation of the Age of Patients

**Figure 2:**
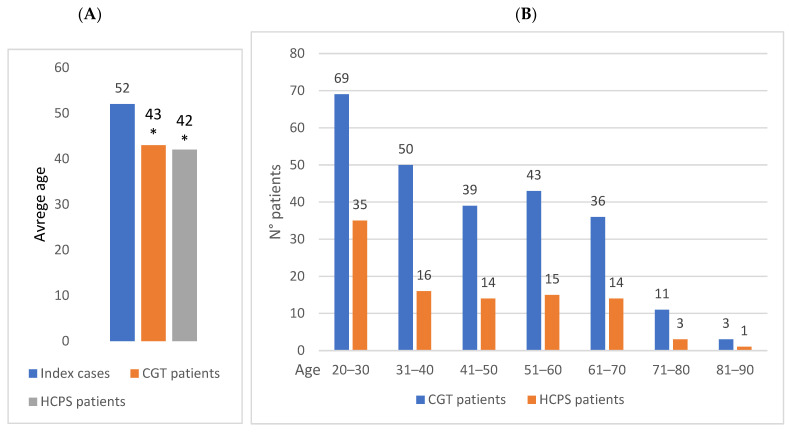
Characterization of the CGT patients and the HCPS patients. (**A**) Comparison of average age with the index cases (years), * Student’s *t*-test, *p* < 0.00001. (**B**) Age breakdown (years).

The examination of patients according to age at the time of CGO reveals that the average age of the index cases was 52 years, that of the CGT patients was 43 years, and that of the HPCS patients was 42 years (Figure 2A). The mean age of both CGT and HPCS patients is significantly higher than that of the index cases (Student’s *t*-test, *p* < 0.00001). The age breakdown is represented in Figure 2B; notably, 52% of HPCS patients were between 20 and 40 years old, and the percentage rises to 66% for patients aged under 50.

### 3.4. Evaluation of Family Relationship Between Patients

**Figure 3:**
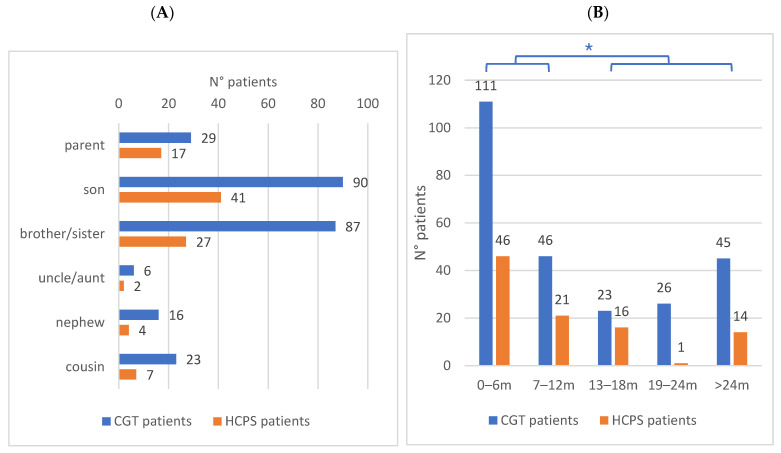
Characterization of the CGT patients and the HCPS carriers. (**A**) Family relationship with the index case. (**B**) Time interval between genetic results (compared to the index case, m = months), * Chi-square test *p* < 0.00001.

The analysis according to family relationships of patients who have requested genetic counseling and testing revealed that 206 patients, corresponding to 83% of the total, are first-degree relatives of the probands, while 45 patients, corresponding to 17%, are second-degree relatives (Table 1). The relationships with the probands allow patients to be divided into seven categories. In particular, the identified 98 HCPS patients were 42% sons, 28% siblings, and 17% parents (Figure 3A).

### 3.5. Evaluation of Time Interval of Information

To estimate the time elapsed for the probe to communicate the outcome of its analysis to the family members and for them to decide to undergo genetic investigation, the study evaluated the interval between the dates of the two related genetic reports. A significantly higher percentage of patients (62.5%, 157/251) underwent cascade testing within a year of the index case report compared to those tested after one year (37.5%, 94/251) (*p* < 0.00001, chi-square test) (Figure 3B). By genetic test, 47% of the HPCS patients were diagnosed within a period of less than six months, and 68% within an interval of less than one year.

### 3.6. Evaluation of the Uptake of CGTs

The percentage of eligible family members who used the cascade test following information from the index cases during the period under review was retrospectively assessed. The total patients/probands ratio was 2.16 (251/116). Considering the patients tested as family members of the index cases with PV identified at the Medical Genetics Unit of Catanzaro, the ratio was 2.32 (58/25). There were no significant differences in the ratios of patients to probands between the two sample groups (chi-square test). The PVs identified by *NGS* at the “Renato Dulbecco” University Hospital during the period 2017-2023 were 40; probands who possessed 25 of these, corresponding to 62.5% of the index cases, generated CGTs in their family members. The first-degree family members of the 40 index cases numbered 186; 58 of these tested for probable hereditary predisposition to the development of tumors (58/186); therefore, the uptake of the test was 31.2%. With reference to the 25 probands who generated the CGT, first-degree family members numbered 108; 53.7% of these tested for probable hereditary predisposition (58/108), and 32.8% (19/58) tested positive for the familial PV.

### 3.7. Evaluation of PVs Identified and Relative Frequencies

The *Sanger* tests performed in the Medical Genetics Unit totaled 257; specifically, 135 in the *BRCA1* gene, 56 in the *BRCA2* gene, 23 in the *TP53* gene, and 43 in other genes. Evaluation of familial genetic variants in carriers identified the presence of 98 pathogenic variants, of which 8 were probably pathogenic variants.

All the germline genetic variants identified in the study are indicated in Table S2, divided by gene localization, along with the relative frequency compared to the total number of patients for the same variant, even across different families in the analyzed cohort.

A total of 50 PVs were identified in the *BRCA1* gene (37% of the analyzed), 28 in the *BRCA2* gene (50%), 5 in the *ATM* gene (56%), 4 in the *MSH6* gene (40%), 3 in the *TP53* gene (13%), and 8 in other genes, as shown in Table S2. The frequency of PVs in the cohort was 0.39 (98/257), while the combined frequency in the *BRCA1/2* genes was 0.41 (78/191).

The most frequently identified genetic variant, found in 12 patients, was the c.4964_4982del variant located in the *BRCA1* gene and considered a *founder* mutation in the Calabrian population [33]. The frequency of this PV was 0.30 (12/40). The highest frequency observed in the *BRCA1* gene was 0.90, with the relative PV being c.1360_1361delAG.

The most frequently identified genetic variant in the *BRCA2* gene, noted in six patients, was the c.8487 + 1G > A variant, with a frequency of 0.40 (6/15). The c.6405_6409del variant, in the same gene, was identified in five patients out of the seven tested, resulting in a frequency of 0.71.

Despite the earlier discovery by the same Medical Genetics Unit of the c.645del PV, located in the *TP53* gene, in a family with Li-Fraumeni syndrome [34], in this study, the most frequently identified genetic variant in the *TP53* gene, found in two patients, was the c.827C>A variant, with a frequency of 0.33 (2/6).

Considering the genes involved in *Lynch* syndrome, the highest frequencies are 0.33 for the likely pathogenic variant c.2291_2297del in the *MSH2* gene and 0.50 for the pathogenic variant c.1610_1613delAGTA in the *MSH6* gene.

## 4. Discussion and Conclusions

The European Commission recommends population-based organized screening programs for cervical, breast, and colorectal cancer. In Italy, the major barriers to effective screening are the incomplete rollout or missed implementation of such programs and, where implemented, the poor participation of the target populations. Italian organized screening programs had a slow scale-up phase, starting from the late 1990s, and are still not completed. The Ministry of Health monitors if the regions guarantee this essential level of assistance through a system of indicators. The achievement of standards of invitation coverage and participation is linked to the funding of the Regional Health Services by the National Health Fund. All Italian regions have now at least partially implemented programs, although in some areas (particularly in the south), there are difficulties in regularly inviting all the target populations. There is a dramatic difference in the spread of organized programs between the north, center, and south of Italy for breast and colorectal cancer screening. Participation on average is low, with the lowest levels in the south, which is also the most deprived area [35]. The Italian National Prevention Plan (PNP) posed the standard to be achieved by Italian regions for the implementation of cervical, breast, and colorectal cancer screening: to invite all of the target populations and to increase the screening uptake up to 50%, 60%, and 50%, respectively, the standard defined by the Essential Levels of Care (called *Livelli Essenziali di Assistenza*, LEA) [36]. Despite considerable debate on how the programs should be implemented, some barriers to extending invitations to all populations, other than a lack of resources, remain. The differences between the north and south, at least for breast and colorectal cancer, are that the areas where the screening programs have never been active are predominantly in the south [35].

The guidelines from the National Institute for Health and Care Excellence indicate the thresholds for testing *BRCA1/2* and other genes involved in hereditary breast and ovarian cancer and *Lynch* syndrome. Particularly, following the identification of a germline transmissible pathogenic variant (PV) in a proband, at-risk family members should be informed about the possibility of undergoing a cascade genetic test (CGT). This could benefit them if they are carriers of the familial PV, through appropriate surveillance programs aimed at early diagnosis, with possible preventive options ranging from intervention on risk factors to surgery for risk reduction (mastectomy and/or oophorectomy) [7,14,15,18,19,20].

The cascade genetic test represents a preventive option for the population, and in this non-homogeneous geographic Italian context, the analysis of patient uptake assumes a relevant role. Some studies have examined the use of cascade testing in hereditary breast and ovarian cancer families in the northern part of Italy [22,37]. There are no studies on the participation of the population of southern Italy in screening programs for the identification of HCPS carriers. The present study focuses on the importance and efficacy of genetic counseling and cascade testing for identifying hereditary cancer predisposition in a cohort of patients from Southern Italy. The aim is to characterize the index cases, the CGT patients, and the carriers of the familial PVs.

The study primarily examines the types of tumors of the probands or the familial PV for which the relatives sought counseling to investigate their carrier status, which determines their suitability for undergoing the genetic test. The outcome shows, as confirmed in other studies [27,28,30], that breast cancer and the *BRCA1/2* genes are perceived as more dangerous, highlighting the need to inform family members and monitor the presence of the PV among them. According to this risk perception, the gender-based analysis reveals that the number of female patients is the most representative; however, the percentage of male patients undergoing CGT increases compared to the index cases. Similar results were obtained by other studies [25,28,30]. Even in a meta-analysis reporting fifty-nine studies among probands, 81.8% are women and 18.2% are men, and female relatives are significantly more likely to complete CGTs than male relatives [38]

Oncological Genetic Counselling (CGO) assumes a central role in selecting patients for genetic testing; however, the disclosure of genetic information between index cases and relatives does not depend solely on the clarity of the information provided but also on pre-existing intrafamilial communication and relationships, family structure, marital status, emotional impact, personal and collective knowledge of tumor pathologies, and various other factors [22,29,30]. For these reasons, it is very important to devote special effort to the correct construction of the pedigree and its revision during the CGTs of family members [17] as well as a personalized approach that accounts for these factors [21,23]. Healthcare providers could play an important role in facilitating intrafamilial communication by giving tailored advice and offering to communicate directly with a patient’s family members; however, no standardized approach has been devised for this purpose. These topics have been investigated mainly in the USA, Australia, and Northern Europe, while there is a relative scarcity of data from other regions of the world, including Southern European countries such as Italy [15,22,37]. In some countries, providers can contact relatives if given permission by the proband [38]. In others, privacy legislation has introduced complexities surrounding the sharing of genetic results with family members and consequently creating a provider-level barrier. In Italy, for instance, healthcare providers are generally prohibited from contacting a proband’s relatives to inform them of the PV in the family or to recommend that they undergo carrier genetic testing [39,40,41]. One of the several strategies to implement the use of CGTs is for the proband to disseminate genetic results with the aid of a family letter that describes the PV, associated cancer risks, and steps for relatives to complete CGT [16,42]. Neither the European nor the British Society for Genetic Medicine guidelines suggest a standard or a template for a family letter, and the way in which it is used in different countries is unclear [43]. However, family letters have demonstrated limited efficacy in facilitating the communication of results [12].

As a result of the numerous problems that interfere with communication between probands and family members, as well as with the execution of CGTs, it is beneficial for the study to explore the characteristics of patients who benefited from genetic information and who requested CGT within the considered time interval. The comparison of the ages of the probands (52 years) and their family members (43 years) reveals a reduction of almost a decade in the average age of patients who sought an assessment of their genetic predisposition to tumor development. Half of the carriers are between 20 and 40 years old. These results align with other studies from the literature [17]. Indeed, in a meta-analysis, the median reported proband age is 51.5 years, and the relative age is 47.4 years [38]. In a multicentric study including Italian women, 12.7% of relatives are under 40 years, 63.5% are between 40 and 60 years, and 23.8% are over 60 years [22].

The analysis based on the family relationships of the patients reveals that 83% of the total CGT is performed by first-degree relatives, particularly the sons of the index cases. In other studies, and in the aforementioned meta-analysis, first-degree relatives are significantly more likely to complete genetic testing compared to second-degree relatives [17,38]. Furthermore, several studies report that probands with children are more likely to complete cascade testing [38] and are most likely to communicate test results to a son [21,26].

The disclosure of genetic information has been described as a process rather than a simple act, occurring over weeks to months. Probands often go through a period of deliberation where they decide what information to disclose, the effects of the disclosure, and the timing of the disclosure [29,30]. A meta-analysis indicates that the proportion of close relatives in each family with a resolved genetic status almost doubled within two years [26,44]. Another study reported an average of 38 months following the genetic testing results of the index cases [17] while others calculated nine months as the median duration of the cascade testing process [25]. Like *Evans DG* et al. [38], in the examined cohort of the present study, the time during which the index cases communicated the presence of the familial PV and the relatives accessed the Medical Genetics Unit for counseling is less than one year for 68% of the patients. Additionally, 47% of CGT patients are diagnosed with a genetic predisposition to develop tumors within a period of fewer than six months from the first genetic report.

First- and second-degree relatives have a 50% and 25% risk of carrying the familial pathogenic variant, respectively [37]; the observed frequency of PVs in the cohort is 39%. This is because the study analyzed second-degree relatives when the closest relative(s) were deceased, and when the indication of PV segregation of the individuals from other facilities was not available, both maternal and paternal branches of the family were included.

The study also evaluates the significant uptake of presymptomatic genetic testing, resulting in 31.2%. Approximately 25% stem from the index cases identified by *NGS* in the same *“Renato Dulbecco” University Hospital*. The total patients/probands ratio is 2.16, a value slightly lower than a study that examined a much larger cohort of patients in the UK [44]. As *Evans DG* et al. noted [44], not all families responded with at least one member, and among those that did, not all potential family members attended. The model that was pursued does not allow for a clear understanding of how much information was shared by the index case. A possible bias may depend on the fact that the test was carried out in another facility or that the family member resides abroad and has not shared the results with relatives, especially those who are more distant.

Despite mounting evidence on the utility of cascade testing, uptake rates among at-risk relatives remain low overall in all countries, although they vary across clinical settings. Some studies report cascade testing uptake rates between 30% and 60%; however, in most of these studies, the uptake rates have been much lower, with only about one-third of probands reporting sharing their test results with their relatives [16,17,25,26,27,28]. The cost–benefit ratio for the healthcare system in performing CGT depends on the number of family members who come forward and, consequently, the number of identified positive family members in the reduction/prevention of new cancer diagnoses to be treated [13]

Moreover, in the overall analysis of cascade testing, the relevant result is the characterization of the PVs identified and the evaluation of their relative frequencies. The frequency of the PVs in the cohort is 0.39, while the combined frequency in the *BRCA1/2* genes is 0.41; particularly, the most frequently identified genetic variant has been the *founder* PV in the Calabrian population, located in the *BRCA1* gene [33].

The current study, through data analysis, confirms and highlights that effective genetic counseling and family communication are crucial to ensure that family members at risk are informed and can undergo measures that prevent advanced tumors. Considering the very encouraging results, we believe that in the Italian population, particularly in southern regions, there is a need for a focus on a proband-mediated approach to communication. The “Renato Dulbecco” University Hospital in Catanzaro did not personally contact the family members of the index cases, according to the guidelines and to the privacy legislation [10,11,14]; however, to reduce the gap between the very high rate of disclosure of genetic testing information to at-risk relatives and the low uptake of genetic testing, future efforts should be directed toward procedures aimed at supporting intra-family communication and improving communication processes between professionals and at-risk relatives [22,37,41]. Results regarding the uptake of cascade testing, lower than those obtained in a study conducted in northern regions [37], indicate that additional efforts are needed for the education of prevention. These differences can be attributed to variations in socio-economic conditions and the inclusion of both highly penetrant and moderately penetrant genes, as well as diverse tumor types, in the analysis.

A higher uptake of genetic testing requires that the National Healthcare System improves its efficacy, efficiency, and equity. Moving on to become interested in health mutation carriers, it will be necessary to define Diagnostic Therapeutic and Care Pathways (PDTA) related to hereditary malignancies, based on a multidisciplinary group supported by the Molecular Tumor Boards (MTB) core team [45]. Organizational and cultural changes are needed to better implement cancer screening in southern Italy, but it has become and will increasingly be essential to analyze the state-of-the-art and periodic temporal trends, as done in this cohort study, to improve strategies and procedures.

## Figures and Tables

**Table 1:** Distribution of the patients involved in the study.

	Index Cases	*CGT Patients*	HCPS Patients
**Total Patients**	116	251 (206 first-degree)	98 (89 first-degree)
***NGS*** **p/o “*R. Dulbecco*”**	25	72 (65 first-grade)	24 (23 first-grade)
***NGS* p/o other facilities**	54	106 (85 first-grade)	45 (40 first-grade)
***Sanger* report**	37	73 (56 first-grade)	29 (26 first-grade)

(Index cases) Probands; (CGT patients) cascade Genetic Test patients; (HCPS patients) Hereditary Cancer Predisposition Syndrome patients; (NGS p/o “R. Dulbecco”) patients that performed NGS analysis at the Medical Genetics Unit of Catanzaro; (NGS p/o other facilities) patients that performed NGS analysis in other facilities; (Sanger report) patients that performed traditional sequencing for the presence of the familial pathogenetic variant.

## Data Availability

The original contributions presented in this study are included in the article/Supplementary Materials. Further inquiries can be directed to the corresponding author(s).

## Supplementary Materials

The following supporting information can be downloaded at: https://www.mdpi.com/article/10.3390/genes16070795/s1, Table S1: Characterization of Cascade Genetic Testing (CGT) Patients and of Index Cases. Table S2: Identified Pathogenic Variants (PVs) and relative frequencies. Figure S1: Flowchart of the Cascade Genetic Testing (CGT) study performed at the Medical Genetics Unit of the “Renato Dulbecco” University Hospital in Catanzaro—Italy. (PV = pathogenetic variant).
