# Supplementary material for: Cascade Genetic Testing for Hereditary Cancer Predisposition: Characterization of Patients in a Catchment Area of Southern Italy
Source: Genes (Basel). 2025 Jun 30;16(7):795. doi: 10.3390/genes16070795 (PMC12295113; doi:10.3390/genes16070795)

Cascade Genetic Testing for Hereditary Cancer Predisposition: Characterization of Patients in a Catchment Area of Southern Italy

Table S1:
Characterization of Cascade Genetic Testing (CGT) Patients and of Index Cases

N.	ID CGT Patient	Age	Gender	Report Date	Family Relationship	ID Index Case/ Ext.Report	Age	Gender	Report Date	Int.Report
**1**	139/17	22	F	Aug-17	son/daughter	153/16	49	F	Jan-17	CGT
**2**	140/17	28	M	Aug-17	son/daughter	153/16	49	F	Jan-17	CGT
**3**	141/17	25	M	Aug-17	son/daughter	153/16	49	F	Jan-17	CGT
**4**	225/17	29	F	Oct-17	son/daughter	277/16	70	F	Mar-17	CGT
**5**	226/17	24	F	Oct-17	son/daughter	277/16	70	F	Mar-17	CGT
**6**	227/17	44	F	Oct-17	brother/sister	277/16	70	F	Mar-17	CGT
**7**	327/17	65	F	Feb-18	brother/sister	ext - NGS	71	F	Jul-17	
**8**	346/17	59	F	Feb-18	uncle/aunt	ext - CGT	59	F	Jan-10	
**9**	418/17	56	F	Apr-18	cousin	ext - NGS	36	F	Nov-16	
**10**	20/18	57	F	Feb-18	parent	ext - NGS	36	F	Nov-16	
**11**	54/18	22	F	Apr-18	son/daughter	ext - NGS	45	F	Jun-09	
**12**	143/18	55	F	May-18	brother/sister	ext - NGS	58	F	Nov-17	
**13**	144/18	47	F	May-18	brother/sister	ext - NGS	58	F	Nov-17	
**14**	145/18	52	F	May-18	brother/sister	ext - NGS	58	F	Nov-17	
**15**	155/18	24	M	May-18	son/daughter	299/17	49	F	Jan-18	NGS
**16**	156/18	19	F	May-18	son/daughter	299/17	49	F	Jan-18	NGS
**17**	179/18	46	F	May-18	brother/sister	299/17	49	F	Jan-18	NGS
**18**	180/18	51	F	May-18	brother/sister	299/17	49	F	Jan-18	NGS
**19**	181/18	44	M	May-18	brother/sister	299/17	49	F	Jan-18	NGS
**20**	216/18	47	F	Jul-18	cousin	227/17	70	F	Oct-17	CGT
**21**	271/18	37	M	Sep-19	brother/sister	ext - CGT	25	F	Nov-18	
**22**	294/18	25	F	Sep-18	brother/sister	ext - MLPA	30	F	Dec-17	
**23**	295/18	32	F	Sep-18	brother/sister	ext - MLPA	30	F	Dec-17	
**24**	296/18	55	F	Aug-18	parent	ext - MLPA	30	F	Dec-17	
**25**	297/18	62	M	Sep-18	parent	ext - MLPA	30	F	Dec-17	
**26**	309/18	30	F	Sep-18	son/daughter	145/18	53	F	May-18	CGT
**27**	689/18	55	F	Jan-19	brother/sister	395/18	51	F	Oct-18	NGS
**28**	690/18	43	F	Jan-19	brother/sister	395/18	51	F	Oct-18	NGS

**29**	691/18	20	F	Jan-19	son/daughter	395/18	51	F	Oct-18	NGS
**30**	694/18	27	M	Mar-19	son/daughter	20/18	50	F	Feb-18	CGT
**31**	695/18	82	F	Mar-19	parent	20/18	50	F	Feb-18	CGT
**32**	70/19	28	M	Mar-19	son/daughter	ext - NGS	60	F	Jan-18	
**33**	71/19	35	F	Mar-19	son/daughter	ext - NGS	60	F	Jan-18	
**34**	89/19	20	F	Mar-19	son/daughter	ext - NGS	43	F	Apr-18	
**35**	106/19	26	M	Mar-19	son/daughter	689/18	56	F	Jan-19	CGT
**36**	128/19	31	F	Mar-19	brother/sister	ext - NGS	37	F	Jan-19	
**37**	129/19	63	M	Mar-19	parent	ext - NGS	37	F	Jan-19	
**38**	160/19	37	F	Mar-20	nephew	ext - NGS	66	F	Dec-18	
**39**	174/19	32	F	May-19	nephew	ext - NGS	50	F	Feb-15	
**40**	300/19	73	F	May-19	parent	395/18	51	F	Oct-18	NGS
**41**	396/19	55	F	Jul-19	brother/sister	ext - NGS	60	F	Jan-18	
**42**	397/19	52	M	Jul-19	brother/sister	ext - NGS	60	F	Jan-18	
**43**	398/19	53	M	Jul-19	brother/sister	ext - NGS	60	F	Jan-18	
**44**	399/19	63	F	Jul-19	brother/sister	ext - NGS	60	F	Jan-18	
**45**	400/19	58	M	Jul-19	brother/sister	ext - NGS	60	F	Jan-18	
**46**	401/19	58	F	Jul-19	brother/sister	ext - NGS	60	F	Jan-18	
**47**	437/19	53	F	Jul-19	brother/sister	ext - NGS	49	F	Dec-18	
**48**	480/19	21	F	Jul-19	son/daughter	ext - CGT	39	F	Jun-05	
**49**	481/19	23	F	Jul-19	son/daughter	ext - CGT	39	F	Jun-05	
**50**	490/19	35	F	Jul-19	cousin	153/16	49	F	Jan-17	CGT
**51**	491/19	18	F	Jul-19	nephew	153/15	49	F	Jan-17	CGT
**52**	492/19	18	F	Jul-19	son/daughter	153/16	49	F	Jan-17	CGT
**53**	538/19	24	F	Oct-19	son/daughter	398/19	53	M	Jul-19	CGT
**54**	634/19	18	F	Oct-19	son/daughter	398/19	53	M	Jul-19	CGT
**55**	635/19	31	M	Oct-19	son/daughter	398/19	53	M	Jul-19	CGT
**56**	636/19	27	M	Oct-19	son/daughter	398/19	53	M	Jul-19	CGT
**57**	668/19	23	F	Oct-19	son/daughter	398/19	53	M	Jul-19	CGT
**58**	750/19	71	F	Dec-19	brother/sister	300/19	74	F	May-19	CGT
**59**	782/19	23	M	Nov-19	brother/sister	ext - NGS	49	F	May-19	
**60**	50/20	65	F	Sep-20	brother/sister	ext - MLPA	60	F	Nov-19	
**61**	99/20	69	M	Mar-20	brother/sister	ext - NGS	66	F	Dec-18	
**62**	113/20	35	F	Sep-20	son/daughter	ext - NGS	56	F	Feb-19	

**63**	398/20	80	M	Sep-20	brother/sister	300/19	74	M	May-19	CGT
**64**	433/20	22	F	Oct-20	son/daughter	ext - CGT	50	F	Oct-16	
**65**	470/20	43	F	Oct-20	brother/sister	ext - CGT	44	F	Mar-20	
**66**	525/20	57	F	Nov-20	brother/sister	ext - NGS	59	M	Jan-20	
**67**	538/20	47	F	Nov-20	cousin	ext - CGT	49	F	Feb-20	
**68**	548/20	27	M	Nov-20	son/daughter	ext - NGS	56	F	Feb-19	
**69**	549/20	31	F	Dec-20	son/daughter	ext - NGS	56	F	Feb-19	
**70**	662/20	34	F	Feb-21	brother/sister	673/19	31	F	Sep-20	NGS
**71**	666/20	35	F	Feb-21	son/daughter	50/20	65	F	Sep-20	CGT
**72**	667/20	40	M	Feb-21	son/daughter	50/20	65	F	Sep-20	CGT
**73**	668/20	25	M	Feb-21	son/daughter	50/20	65	F	Sep-20	CGT
**74**	669/20	38	F	Feb-21	son/daughter	50/20	65	F	Sep-20	CGT
**75**	121/21	61	M	Apr-21	brother/sister	ext - CGT	55	F	Jul-20	
**76**	122/21	49	M	Apr-21	brother/sister	ext - CGT	55	F	Jul-20	
**77**	126/21	84	F	Apr-21	parent	ext - CGT	55	F	Jul-20	
**78**	173/21	72	M	Apr-21	brother/sister	ext - NGS	70	M	Dec-20	
**79**	174/21	44	M	Apr-21	nephew	ext - NGS	70	M	Dec-20	
**80**	175/21	30	M	Apr-21	nephew	ext - NGS	70	M	Dec-20	
**81**	199/21	32	M	Apr-21	nephew	764/19	67	M	Apr-20	NGS
**82**	200/21	37	M	Apr-21	nephew	764/19	67	M	Apr-20	NGS
**83**	206/21	61	F	Mar-21	brother/sister	ext - NGS	59	M	Jan-20	
**84**	221/21	38	M	Apr-21	cousin	ext - NGS	36	F	Sep-20	
**85**	222/21	49	M	Apr-21	uncle/aunt	ext - NGS	36	F	Sep-20	
**86**	224/21	79	F	Apr-21	parent	ext - CGT	44	F	Oct-20	
**87**	233/21	66	F	Apr-21	brother/sister	ext - CGT	52	F	Aug-20	
**88**	234/21	31	M	Apr-21	son/daughter	ext - CGT	52	F	Aug-20	
**89**	235/21	29	F	Apr-21	son/daughter	ext - CGT	52	F	Aug-20	
**90**	236/21	25	M	Apr-21	son/daughter	ext - CGT	52	F	Aug-20	
**91**	303/21	54	M	Jul-21	brother/sister	ext - CGT	52	F	Aug-20	
**92**	389/21	30	F	Jul-21	nephew	ext - CGT	59	F	Jun-20	
**93**	393/21	32	F	Jul-21	nephew	221/21	49	M	Apr-21	CGT
**94**	394/21	64	F	Jul-21	brother/sister	221/21	49	M	Apr-21	CGT
**95**	395/21	41	M	Jul-21	nephew	221/21	49	M	Apr-21	CGT
**96**	397/21	35	M	Jul-21	son/daughter	233/21	66	F	Apr-21	CGT

**97**	398/21	80	F	Jul-21	uncle/aunt	233/21	66	F	Apr-21	CGT
**98**	441/21	70	M	Jul-21	brother/sister	ext - NGS	68	F	Dec-19	
**99**	460/21	36	F	Jul-21	nephew	764/19	67	M	Oct-19	NGS
**100**	478/21	37	M	Jul-21	nephew	764/19	67	M	Oct-19	NGS
**101**	491/21	67	F	Sep-21	parent	ext - NGS	44	F	Mar-18	
**102**	562/21	72	M	Sep-21	parent	223/21	37	F	May-21	NGS
**103**	616/21	34	M	Sep-21	brother/sister	223/21	37	F	May-21	NGS
**104**	617/21	38	M	Sep-21	brother/sister	223/21	37	F	May-21	NGS
**105**	631/21	33	M	Sep-21	son/daughter	ext - NGS	60	F	Feb-20	
**106**	738/21	52	M	Nov-21	son/daughter	432/21	81	F	Sep-21	NGS
**107**	764/21	42	M	Nov-21	brother/sister	763/21	35	F	Nov-21	CGT
**108**	782/21	39	M	Dec-21	brother/sister	ext - NGS	42	F	Jul-21	
**109**	783/21	60	F	Dec-21	parent	ext - NGS	42	F	Jul-21	
**110**	784/21	64	M	Dec-21	parent	ext - NGS	42	F	Jul-21	
**111**	835/21	29	F	Dec-21	son/daughter	99/21	69	M	Sep-21	NGS
**112**	836/21	46	F	Dec-21	son/daughter	99/21	69	M	Sep-21	NGS
**113**	837/21	36	F	Dec-21	son/daughter	99/21	69	M	Sep-21	NGS
**114**	946/21	68	F	Jan-22	brother/sister	ext - CGT	71	F	Oct-21	
**115**	993/21	57	F	Jan-22	parent	223/21	37	F	May-21	NGS
**116**	13/22	35	F	Jan-22	brother/sister	ext - NGS	37	F	Apr-21	
**117**	13/22	35	F	Jan-22	cousin	488/21	31	F	Sep-21	NGS
**118**	14/22	65	F	Jan-22	parent	ext - NGS	37	F	Apr-21	
**119**	14/22	66	F	Jan-22	uncle/aunt	488/21	31	F	Sep-21	NGS
**120**	15/22	70	M	Jan-22	parent	ext - NGS	37	F	Apr-21	
**121**	23/22	34	F	Feb-22	cousin	ext - NGS	37	F	Apr-21	
**122**	23/22	35	F	Feb-22	brother/sister	488/21	31	F	Sep-21	NGS
**123**	87/22	59	M	Feb-22	brother/sister	279/21	54	F	Jun-21	NGS
**124**	170/22	27	M	Feb-22	cousin	ext - NGS	37	F	Apr-21	
**125**	170/22	27	M	Feb-22	brother/sister	488/21	31	F	Sep-21	NGS
**126**	192/22	33	F	Mar-22	nephew	ext - NGS	30	F	Aug-04	
**127**	301/22	31	F	Apr-22	brother/sister	ext - NGS	35	F	Aug-21	
**128**	399/22	57	M	May-22	brother/sister	223/21	37	F	Jan-22	CGT
**129**	400/22	61	M	May-22	brother/sister	223/21	37	F	Jan-22	CGT
**130**	418/22	61	F	May-22	uncle/aunt	ext - NGS	38	F	Jun-18	

**131**	552/22	46	F	Jun-22	brother/sister	69/22	49	F	Mar-22	NGS
**132**	553/22	52	M	Jun-22	brother/sister	69/22	49	F	Mar-22	NGS
**133**	554/22	43	M	Jun-22	brother/sister	69/22	49	F	Mar-22	NGS
**134**	555/22	34	F	Jun-22	son/daughter	69/22	49	F	Mar-22	NGS
**135**	556/22	26	F	Jun-22	son/daughter	69/22	49	F	Mar-22	NGS
**136**	582/22	43	M	Jun-22	son/daughter	99/21	69	M	Sep-21	NGS
**137**	583/22	51	F	Jun-22	nephew	ext - NGS	51	F	May-21	
**138**	584/22	57	F	Jun-22	nephew	ext - NGS	51	F	May-21	
**139**	607/22	44	F	Jul-22	brother/sister	ext - NGS	42	F	Sep-13	
**140**	610/22	27	M	Jul-22	son/daughter	ext - CGT	58	F	Jul-20	
**141**	648/22	42	M	Jul-22	son/daughter	713/21	62	F	Sep-21	NGS
**142**	704/22	46	F	Aug-22	brother/sister	ext - NGS	41	F	Jan-15	
**143**	722/22	68	F	Jul-22	parent	428/22	36	F	Jun-22	NGS
**144**	723/22	39	F	Jul-22	brother/sister	428/22	36	F	Jun-22	NGS
**145**	724/22	74	M	Jul-22	parent	428/22	36	F	Jun-22	NGS
**146**	729/22	26	F	Aug-22	son/daughter	223/21	37	F	May-22	CGT
**147**	730/22	29	F	Aug-22	son/daughter	223/21	37	F	May-22	CGT
**148**	731/22	41	F	Aug-22	brother/sister	ext - NGS	39	F	Jun-22	
**149**	732/22	61	F	Aug-22	parent	ext - NGS	39	F	Jun-22	
**150**	735/22	35	F	Aug-22	brother/sister	ext - NGS	37	F	Aug-20	
**151**	812/22	18	F	Oct-22	son/daughter	607/22	44	F	Jul-22	CGT
**152**	836/22	46	F	Oct-22	son/daughter	ext - NGS	69	M	Feb-20	
**153**	837/22	36	M	Oct-22	son/daughter	ext - NGS	69	M	Feb-20	
**154**	948/22	53	F	Nov-22	son/daughter	444/22	75	F	Jun-22	NGS
**155**	949/22	51	F	Nov-22	son/daughter	444/22	75	F	Jun-22	NGS
**156**	992/22	67	M	Nov-22	brother/sister	ext - CGT	54	F	Sep-21	
**157**	1015/22	38	F	Jan-23	son/daughter	276/22	63	F	Sep-22	NGS
**158**	1030/22	58	F	Jan-23	cousin	ext - NGS	62	F	Oct-21	
**159**	1031/22	54	F	Jan-23	cousin	ext - NGS	62	F	Oct-21	
**160**	1032/22	68	F	Jan-23	cousin	ext - NGS	62	F	Oct-21	
**161**	1083/22	18	M	Jan-23	son/daughter	732//22	61	F	Aug-22	CGT
**162**	1119/22	24	F	Jan-23	son/daughter	ext - NGS	51	F	Sep-22	
**163**	1120/22	29	F	Jan-23	son/daughter	ext - NGS	51	F	Sep-22	
**164**	1121/22	26	F	Jan-23	son/daughter	ext - NGS	51	F	Sep-22	

**165**	1163/22	60	M	Jan-23	parent	ext - NGS	35	F	Dec-21	
**166**	1164/22	26	F	Jan-23	brother/sister	ext - NGS	35	F	Dec-21	
**167**	1165/22	28	M	Jan-23	brother/sister	ext - NGS	35	F	Dec-21	
**168**	1166/22	60	F	Jan-23	parent	ext - NGS	35	F	Dec-21	
**169**	1173/22	62	M	May-23	brother/sister	ext - NGS	60	M	Mar-09	
**170**	16/23	23	F	Jan-23	son/daughter	20/18	58	F	Feb-18	CGT
**171**	17/23	26	F	Jan-23	son/daughter	164/21	70	F	Jul-21	NGS
**172**	73/23	48	F	Feb-23	brother/sister	ext - NGS	35	F	Oct-22	
**173**	74/23	68	F	Feb-23	parent	ext - NGS	35	F	Oct-22	
**174**	119/23	51	F	Feb-23	brother/sister	ext - NGS	48	F	Mar-21	
**175**	164/23	63	F	Feb-23	parent	ext - NGS	37	F	Sep-21	
**176**	165/23	53	F	Feb-23	brother/sister	ext - NGS	47	F	Jul-22	
**177**	246/23	40	M	Mar-23	son/daughter	ext - NGS	30	F	Aug-04	
**178**	247/23	49	M	Mar-23	son/daughter	ext - NGS	30	F	Aug-04	
**179**	251/23	71	F	Mar-23	parent	540/22	51	F	Oct-22	NGS
**180**	252/23	46	M	Mar-23	brother/sister	540/22	51	F	Oct-22	NGS
**181**	253/23	51	F	Mar-23	brother/sister	540/22	51	F	Oct-22	NGS
**182**	254/23	38	F	Mar-23	brother/sister	540/22	51	F	Oct-22	NGS
**183**	374/23	68	M	Apr-23	parent	ext - NGS	40	F	Jan-23	
**184**	377/23	54	M	Apr-23	brother/sister	ext - NGS	36	F	Oct-17	
**185**	505/23	19	F	May-23	son/daughter	ext - NGS	45	F	Nov-17	
**186**	512/23	31	F	May-23	son/daughter	ext - NGS	62	F	Jan-23	
**187**	513/23	38	M	May-23	son/daughter	ext - NGS	62	F	Jan-23	
**188**	596/23	42	F	Jun-23	nephew	ext - CGT	60	F	May-21	
**189**	715/23	46	F	Jun-23	cousin	ext - NGS	39	F	Mar-14	
**190**	745/23	25	F	Jun-23	son/daughter	ext - NGS	43	F	Jun-15	
**191**	783/23	37	M	Jun-23	son/daughter	1023/22	62	F	Apr-23	NGS
**192**	784/23	43	F	Jun-23	son/daughter	1023/22	62	F	Apr-23	NGS
**193**	785/23	65	F	Jun-23	brother/sister	1023/22	62	F	Apr-23	NGS
**194**	904/23	62	F	Jul-23	cousin	20/18	58	F	Feb-18	CGT
**195**	905/23	38	M	Jul-23	cousin	20/18	58	F	Feb-18	CGT
**196**	906/23	28	F	Jul-23	cousin	20/18	58	F	Feb-18	CGT
**197**	913/23	22	M	Jul-23	son/daughter	256/22	47	F	May-22	NGS
**198**	973/23	62	F	Sep-23	cousin	ext - NGS	59	F	Mar-23	

**199**	974/23	60	F	Sep-23	cousin	ext - NGS	59	F	Mar-23	
**200**	975/23	60	F	Sep-23	cousin	ext - NGS	59	F	Mar-23	
**201**	1000/23	30	F	Sep-23	son/daughter	943/21	51	F	Feb-22	NGS
**202**	1001/23	24	M	Sep-23	son/daughter	943/21	51	F	Feb-22	NGS
**203**	1035/23	22	F	Sep-23	son/daughter	ext - NGS	56	F	Mar-20	
**204**	1036/23	21	F	Sep-23	son/daughter	ext - NGS	56	F	Mar-20	
**205**	1093/23	37	M	Sep-23	brother/sister	1057/22	32	M	Apr-23	NGS
**206**	1139/23	54	F	Nov-23	brother/sister	ext - NGS	42	F	Dec-21	
**207**	1221/23	60	F	Jan-24	son/daughter	ext - CGT	53	F	Jun-23	
**208**	1224/23	28	F	Nov-23	son/daughter	ext - NGS	53	F	Mar-23	
**209**	1295/23	67	F	Nov-23	parent	263/23	37	F	Jul-23	NGS
**210**	1296/23	24	F	Nov-23	brother/sister	263/23	37	F	Jul-23	NGS
**211**	1297/23	42	M	Nov-23	brother/sister	263/23	37	F	Jul-23	NGS
**212**	1298/23	66	M	Nov-23	parent	263/23	37	F	Jul-23	NGS
**213**	1315/23	58	F	Nov-23	parent	1189/22	33	F	Apr-23	NGS
**214**	1332/23	59	F	Dec-23	son/daughter	322/23	77	F	Jul-23	NGS
**215**	1400/23	44	M	Jan-24	son/daughter	1095/22	66	F	Apr-23	NGS
**216**	1401/23	22	F	Dec-23	son/daughter	607/22	44	F	Jul-22	CGT
**217**	1402/23	73	F	Dec-23	brother/sister	ext - NGS	60	M	Mar-09	
**218**	1421/23	50	F	Dec-23	brother/sister	ext - NGS	49	F	Dec-20	
**219**	14/24	25	M	Jan-24	son/daughter	704/22	56	F	Aug-22	CGT
**220**	16/24	31	F	Mar-24	son/daughter	ext - NGS	63	M	Nov-23	
**221**	17/24	34	F	Mar-24	son/daughter	ext - NGS	63	M	Nov-23	
**222**	62/24	30	M	Jan-24	cousin	1057/22	30	M	Apr-23	NGS
**223**	66/24	69	F	Jan-24	brother/sister	ext - NGS	60	M	Mar-09	
**224**	71/24	45	F	Jun-24	cousin	ext - CGT	42	F	Apr-18	
**225**	106/24	62	F	Jan-24	brother/sister	ext - NGS	42	F	Dec-21	
**226**	132/24	30	F	Feb-24	son/daughter	322/23	77	F	Dec-23	CGT
**227**	218/24	33	F	Feb-24	brother/sister	ext - NGS	50	F	Aug-23	
**228**	219/24	46	M	Feb-24	brother/sister	ext - NGS	50	F	Aug-23	
**229**	251/24	37	M	Mar-24	son/daughter	276/22	63	F	Sep-22	NGS
**230**	300/24	56	F	Mar-24	brother/sister	832/23	52	F	Nov-23	NGS
**231**	326/24	61	F	Mar-24	brother/sister	ext - NGS	63	M	Nov-23	
**232**	327/24	65	M	Mar-24	brother/sister	ext - NGS	63	M	Nov-23	

**233**	365/24	49	F	May-24	son/daughter	ext - NGS	78	F	Dec-23	
**234**	369/24	47	F	May-24	son/daughter	ext - NGS	78	F	Dec-23	
**235**	368/24	47	F	May-24	son/daughter	ext - NGS	78	F	Dec-23	
**236**	417/24	37	M	May-24	brother/sister	840/23	35	F	Nov-23	NGS
**237**	418/24	30	F	May-24	brother/sister	840/23	35	F	Nov-23	NGS
**238**	419/24	28	F	May-24	brother/sister	840/23	35	F	Nov-23	NGS
**239**	420/24	71	F	May-24	parent	840/23	35	F	Nov-23	NGS
**240**	421/24	59	F	May-24	parent	840/23	35	F	Nov-23	NGS
**241**	422/24	42	F	May-24	son/daughter	ext - NGS	67	F	Dec-21	
**242**	423/24	74	F	May-24	uncle/aunt	ext - NGS	63	F	May-19	
**243**	197/24	58	F	May-24	brother/sister	ext - NGS	56	F	Nov-23	
**244**	452/24	23	M	Jun-24	son/daughter	230/24	56	M	Feb-21	CGT
**245**	453/24	20	F	Jun-24	son/daughter	230/24	56	M	Feb-21	CGT
**246**	492/24	19	F	May-24	son/daughter	94/23	57	M	May-22	NGS
**247**	493/24	51	M	May-24	brother/sister	94/23	57	M	May-22	NGS
**248**	538/24	18	M	May-24	son/daughter	94/23	57	M	May-22	NGS
**249**	534/24	50	M	Jun-24	cousin	ext - NGS	30	F	Aug-04	
**250**	535/24	19	F	Jun-24	cousin	ext - NGS	30	F	Aug-04	
**251**	596/24	46	F	Jun-24	cousin	ext - CGT	42	F	Apr-18	

Cascade Genetic Testing for Hereditary Cancer Predisposition: Characterization of Patients in a Catchment Area of Southern Italy

Table S2.
Identified Pathogenic Variants (PVs) and relative frequencies.

Gene	PV	Classification	N° positive	N° families	N° test	Freq. *BRCA1/2*	Freq. *BRCA1*	Freq. *BRCA2*	Freq. PV spec.
BRCA1	c.4964_4982del	Pathogenic	12	23	40	0,06	0,09		0,30
BRCA1	c.1360_1361delAG	Pathogenic	9	3	10	0,05	0,07		0,90
BRCA1	c.5266dupC	Pathogenic	6	5	7	0,03	0,04		0,86
BRCA1	c.181T>G	Pathogenic	6	8	18	0,03	0,04		0,33
BRCA1	c.514del	Pathogenic	3	3	8	0,02	0,02		0,38
BRCA1	c.(5193+1_5194-1)_(5277+1_5278-1)del	Pathogenic	3	1	4	0,02	0,02		0,75
BRCA1	c.134+2T>G	Pathogenic	2	2	6	0,01	0,01		0,33
BRCA1	c.5123C>A	Pathogenic	2	2	8	0,01	0,01		0,25
BRCA1	c.4484G>T	Pathogenic	2	2	3	0,01	0,01		0,67
BRCA1	c.68_69del	Pathogenic	2	2	3	0,01	0,01		0,67
BRCA1	c.1870G>T	Pathogenic	1	1	1	0,01	0,01		1,00
BRCA1	c.4327C>T	Pathogenic	1	1	2	0,01	0,01		0,50
BRCA1	c.1266T>A	Pathogenic	1	2	2	0,01	0,01		0,50
BRCA2	c.8487+1G>A	Pathogenic	6	3	15	0,03		0,11	0,40
BRCA2	c.6405_6409del	Pathogenic	5	6	7	0,03		0,09	0,71
BRCA2	c.4284dupT	Pathogenic	3	1	3	0,02		0,05	1,00
BRCA2	c.2657del	Pathogenic	3	2	6	0,02		0,05	0,50
BRCA2	c.7008-2A>T	Pathogenic	2	3	9	0,01		0,04	0,22
BRCA2	c.6509_6513delinsGTC	Pathogenic	2	1	2	0,01		0,04	1,00
BRCA2	c.5073dup	Pathogenic	2	1	2	0,01		0,04	1,00
BRCA2	c.7805+1G>A	Pathogenic	2	1	2	0,01		0,04	1,00
BRCA2	c.631G>A	Pathogenic	1	3	9	0,01		0,02	0,11
BRCA2	c.1670T>G	Pathogenic	1	2	3	0,01		0,02	0,33
BRCA2	c.5851_5854del	Pathogenic	1	1	1	0,01		0,02	1,00
MSH6	c.1610_1613delAGTA	Pathogenic	3	3	6				0,50
MSH6	c.1A>T	Likely Pathogenic	1	1	2				0,50
MSH2	c.2291_2297del	Likely Pathogenic	1	1	3				0,33

ATM	c.8977C>T	Pathogenic	2	1	2				1,00
ATM	c.7456C>T	Pathogenic	2	1	2				1,00
ATM	c.8151+1G>T	Likely Pathogenic	1	1	4				0,25
CHEK2	c.846+1G>C	Likely Pathogenic	1	1	4				0,25
MLH1	c.208-3C>G	Likely Pathogenic	2	1	2				1,00
TP53	c.827C>A	Likely Pathogenic	2	1	6				0,33
TP53	c.451C>G	Likely Pathogenic	1	2	8				0,13
MITF	c.1273G>A	Pathogenic	1	1	1				1,00
APC	c.1863_1866del	Pathogenic	1	1	2				0,50
BRIP1	c.2392C>T	Pathogenic	1	1	1				1,00
RAD51C	c.577C>T	Pathogenic	1	1	4				0,25

Cascade Genetic Testing for Hereditary Cancer Predisposition: Characterization of Patients in a Catchment Area of Southern Italy

Figure S1:
Flowchart of the Cascade Genetic Testing (CGT) study performed at the Medical Genetics Unit of the "Renato Dulbecco" University Hospital in Catanzaro– Italy.
(PV=pathogenetic variant)

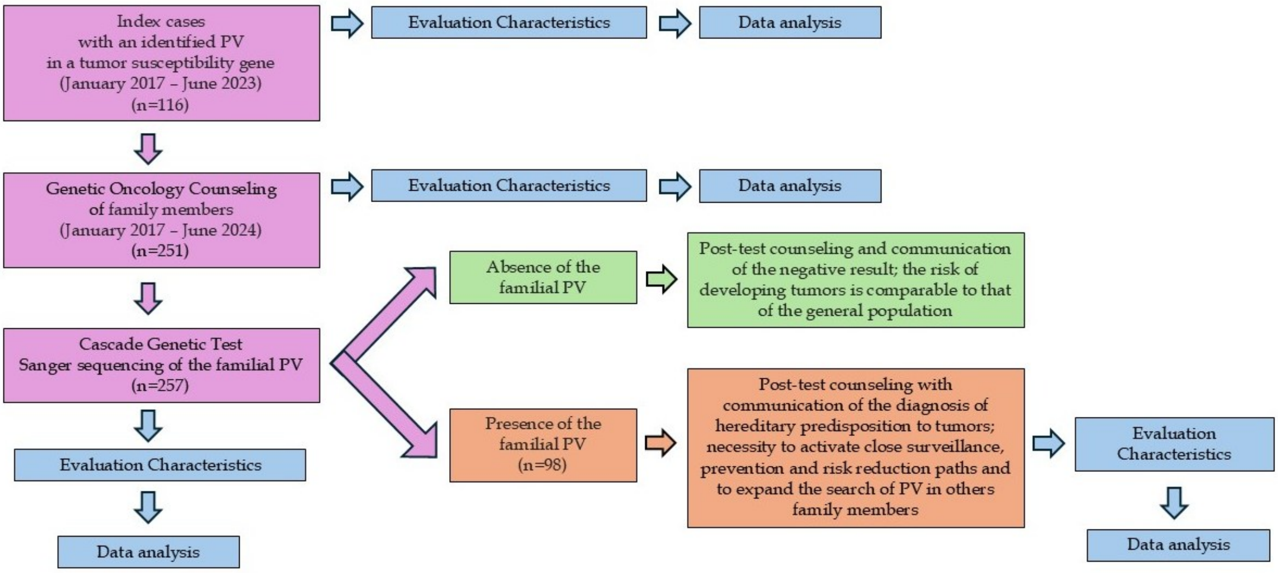

Supplement: Supplementary file 1 [file genes-16-00795-s001.zip › genes-3688659-supplementary.pdf]
